# The mediating role of sleep quality in the association between inflammatory disease activity and health-related quality of life in rheumatoid arthritis

**DOI:** 10.3389/fmed.2026.1797652

**Published:** 2026-03-16

**Authors:** Zeynel Abidin Akar, Dilan Yıldırım, Ömer Karakoyun, Kadir Kaya, İbrahim Batmaz, Serda Em, Mehmet Karakoç

**Affiliations:** 1 Division of Rheumatology, Department of Physical Therapy and Rehabilitation, Faculty of Medicine, Dicle University, Diyarbakır, Türkiye; 2 Department of Physical Therapy and Rehabilitation, Faculty of Medicine, Dicle University, Diyarbakır, Türkiye; 3 Department of Dermatology and Venereology, Faculty of Medicine, Dicle University, Diyarbakır, Türkiye; 4 Department of Dermatology and Venereology, Faculty of Medicine, İstinye University, Istanbul, Türkiye

**Keywords:** health-related quality of life, inflammation, mediation analysis, rheumatoid arthritis, sleep quality, vitamin D deficiency

## Abstract

**Background:**

Sleep disturbance is highly prevalent in rheumatoid arthritis (RA) and is a key determinant of patient-reported outcomes. However, the mechanistic pathways linking systemic inflammation and metabolic dysregulation, defined in this study as insulin resistance and adverse cardiometabolic indices, and sleep quality remain poorly defined. This study aimed to investigate the interplay between inflammatory/metabolic markers and sleep quality, and to determine whether sleep quality mediates the relationship between disease activity by DAS28-ESR and health-related quality of life.

**Methods:**

In this cross-sectional study, 128 patients with RA and 115 healthy controls were evaluated. Systemic inflammation and metabolic stress were characterized using ESR, CRP, neutrophil-to-lymphocyte ratio, platelet-to-lymphocyte ratio, and indices including the TyG index and cardiometabolic index. Sleep was assessed via the Pittsburgh Sleep Quality Index and HRQoL via the Short Form-36. Mediation analyses (5,000 bootstrap resamples), adjusted for age, sex, smoking status, education level, and disease duration, and sensitivity analyses were performed to quantify the indirect effects of sleep quality on HRQoL domains.

**Results:**

RA patients exhibited significantly higher inflammatory and metabolic burden, markedly poorer sleep quality, and lower Vitamin D levels than controls (all *p* < 0.001). DAS28-ESR was the strongest independent predictor of poor sleep (*β* = 0.534, *p* < 0.001), while Vitamin D was an independent predictor of better sleep (*β* = −0.173, *p* = 0.002). Mediation analyses revealed that sleep quality may significantly mediate the association between disease activity and life quality, accounting for 24.4% of the effect of DAS28-ESR on Mental Health (Indirect Effect = −1.42; 95% CI − 2.38 to −0.62) and 23.6% on General Health. Sensitivity analyses confirmed these mediating effects were robust across BMI and gender subgroups.

**Conclusion:**

Sleep quality may represent a critical mechanistic link through which inflammatory disease activity translates into impaired HRQoL in RA. These findings suggest that nearly one-quarter of the disease’s psychological burden may be statistically mediated through sleep disruption. Integrating systematic sleep assessment and Vitamin D optimization into routine care may help mitigate the patient-perceived disease burden. Given the cross-sectional design, these findings should be interpreted as evidence of statistical mediation rather than confirmed causal pathways.

## Highlights

- What is already known?
- Sleep disturbances are highly prevalent in patients with rheumatoid arthritis (RA), yet their quantitative contribution to the relationship between disease activity and patient-reported health outcomes remains incompletely defined.
- What does this study add?
- This study demonstrates that sleep quality may represent a key mediating pathway, explaining approximately 25% of the association between RA disease activity and patient-reported mental and general health outcomes in a cross-sectional model.
- How might this look in clinical practice?
- These findings suggest that achieving clinical remission alone may not fully address the patient-perceived disease burden if sleep disturbances persist, highlighting the potential value of incorporating sleep assessment and management into treat-to-target (T2T) strategies in RA, pending prospective validation.

## Introduction

Rheumatoid arthritis (RA) is a chronic, systemic autoimmune disorder affecting approximately 0.5–1% of the global population, with a markedly higher prevalence in women (1). Beyond progressive synovial inflammation and joint destruction, RA is increasingly recognized as a multi-systemic disease frequently accompanied by extra-articular manifestations, including cardiovascular complications, metabolic dysregulation—particularly insulin resistance and adverse cardiometabolic alterations—, and profound sleep disturbances (2). These interconnected factors create a complex clinical landscape that contributes to increased morbidity and mortality, as well as substantial reductions in health-related quality of life (HRQoL) (3). Persistent systemic inflammation, together with adverse metabolic profiles, is considered a major driver of the “accelerated aging” and heightened cardiovascular risk observed in RA patients (4). Sleep disturbances, commonly reported in individuals with RA, further exacerbate fatigue, amplify pain perception, and impair both physical and mental domains of HRQoL (5). Therefore, elucidating the pathways through which inflammatory activity and metabolic factors intersect with sleep quality may provide important insights into disease burden beyond articular involvement. Such insights are critical for developing a holistic management strategy that extends beyond joint-focused metrics to optimize patient-centered outcomes in RA (6).

Chronic pain, debilitating fatigue, and reduced functional capacity constitute the core clinical challenges of RA, collectively imposing a substantial burden on patients’ physical and mental well-being (7). Sleep disturbances—often under-recognized in routine clinical practice—are frequently reported among individuals with RA (8). Compared with healthy controls, patients with RA commonly experience shorter sleep duration, increased nocturnal awakenings, and markedly poorer subjective sleep quality (9). These impairments are not merely secondary symptoms; they appear to function as bi-directional contributors to disease burden, closely associated with heightened pain sensitivity, exacerbated fatigue, and reduced treatment adherence, thereby further compromising health-related quality of life (HRQoL) (10). Although the relationship between joint inflammation and sleep disruption is well documented, the contribution of systemic metabolic indices—such as the triglyceride–glucose (TyG) index (a surrogate marker of insulin resistance) and cardiometabolic index—remains poorly understood. Moreover, whether sleep quality acts as a statistical mediating pathway through which systemic inflammatory burden translates into diminished HRQoL has not been clearly established. Recognizing and targeting sleep disturbances is therefore critical, as they may represent a potentially modifiable mechanism to improve patient-centered outcomes in RA.

Inflammatory markers, including erythrocyte sedimentation rate (ESR), C-reactive protein (CRP), and ferritin, not only reflect disease activity but also provide an index of systemic inflammatory burden in RA. Emerging evidence indicates that elevated levels of these markers are associated with poorer sleep quality, underscoring the pervasive impact of inflammation beyond joint pathology (11). Concurrently, vitamin D deficiency is highly prevalent in RA and has garnered increasing attention due to its pleiotropic effects on immune regulation, sleep architecture, and mood (12). Lower vitamin D levels have been linked to higher disease activity and disrupted sleep patterns, suggesting a pathophysiological interplay among systemic inflammation, vitamin D status, and impaired HRQoL (13). Additionally, metabolic dysregulation—particularly insulin resistance and adverse cardiometabolic profiles captured by indices such as the TyG index and cardiometabolic index (CMI)—has emerged as a potential contributor to the systemic burden of RA, although its relationship with sleep remains incompletely understood. Collectively, these findings highlight sleep quality as a potential statistical mediator through which inflammatory and metabolic factors may be associated with patient-centered outcomes.

Metabolic dysfunction represents another important and multifaceted contributor to the overall disease burden in RA (14). Specifically, metabolic abnormalities—including obesity, insulin resistance, and increased visceral adiposity—may exacerbate disease activity via pro-inflammatory pathways while also potentially impairing sleep quality and physical functioning (15). Recently, emerging biomarkers and composite indices, such as the TyG index, waist-to-height ratio, and neutrophil-to-lymphocyte ratio (NLR), have been increasingly utilized to capture the complex interplay between metabolic stress and systemic inflammation (16). These metabolic disturbances may act in concert with inflammatory burden and vitamin D deficiency, contributing to sleep disruption and, consequently, to reduced health-related quality of life (HRQoL).

Despite these documented associations, the specific pathways linking inflammation, metabolic dysregulation, and sleep quality remain incompletely understood. In particular, it is unclear whether sleep quality may serve as a primary mediator connecting the combined burden of inflammatory activity and metabolic stress to impaired HRQoL. Accordingly, this study aimed to compare inflammatory, metabolic, and sleep-related parameters between RA patients and healthy controls, identify independent predictors of sleep quality among clinical and biochemical variables, and evaluate whether sleep quality mediates the association between disease activity and HRQoL. We hypothesized that poor sleep quality would be independently influenced by both inflammatory and metabolic indices and could serve as a key mediator between disease activity and the physical and mental components of HRQoL.

## Materials and methods

### Study population and ethical approval

This cross-sectional study was conducted through a retrospective analysis of institutional data from patients with rheumatoid arthritis (RA) and age- and sex-matched healthy controls. The RA cohort included 128 patients who fulfilled the 2010 ACR/EULAR classification criteria (17). Healthy controls were recruited from the same institution and had no documented history of autoimmune, inflammatory, or chronic systemic diseases.

Exclusion criteria were applied uniformly to both groups to minimize potential confounding factors. Individuals were excluded if they had coexisting autoimmune or systemic inflammatory disorders (e.g., systemic lupus erythematosus, spondyloarthritis), active malignancy or acute infections within the preceding month, acute metabolic crises (e.g., uncontrolled diabetes mellitus or thyroid storm), or a known history of primary sleep disorders (e.g., obstructive sleep apnea) or recent use of medications for insomnia (e.g., hypnotics) within the last 2 weeks. No patients underwent objective sleep studies such as polysomnography, nor were any receiving CPAP therapy. Sleep quality was assessed exclusively via the Pittsburgh Sleep Quality Index (PSQI), a validated self-reported instrument.

**Figure 1:**
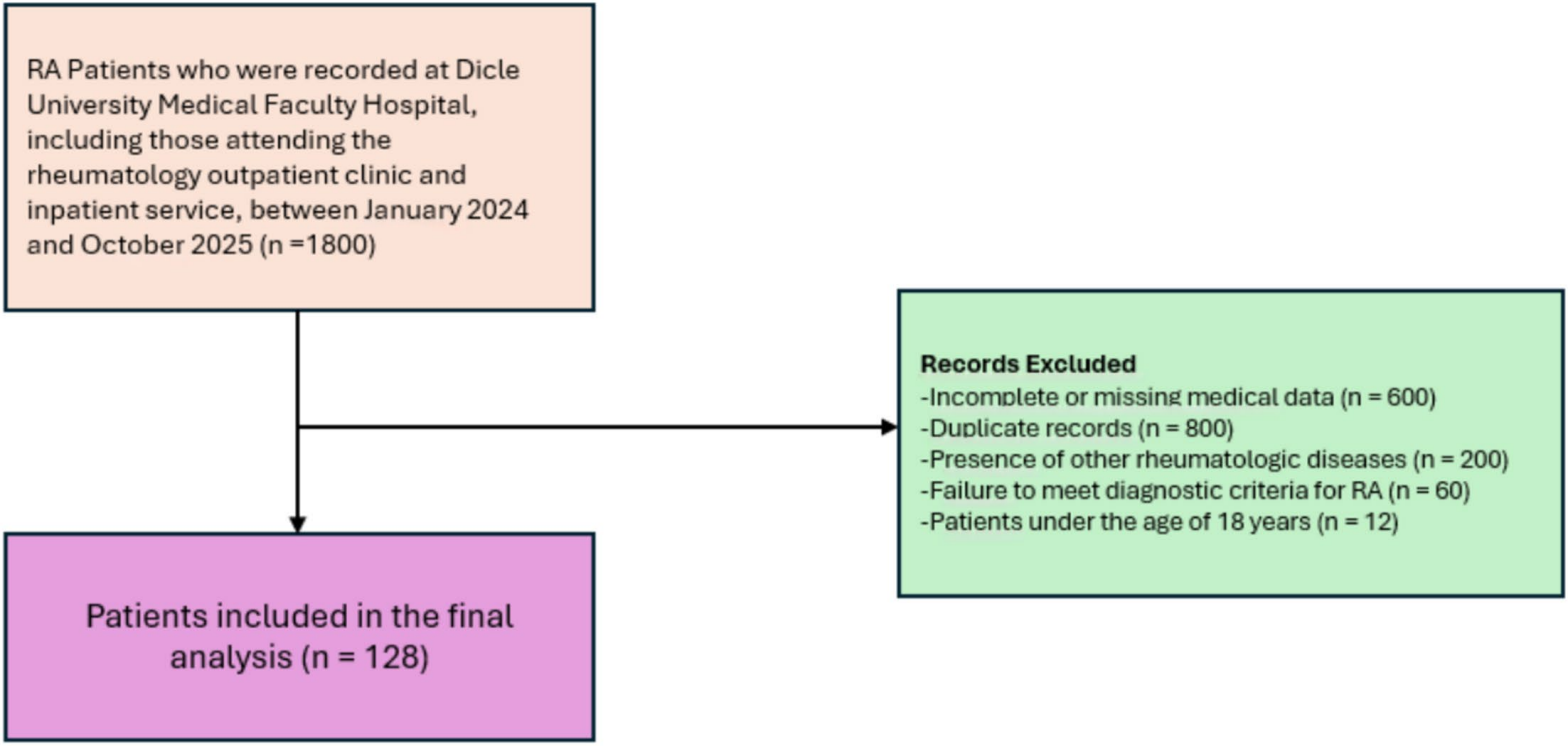
Flowchart of the participant selection process. This flowchart illustrates the systematic exclusion criteria applied to the initial patient pool at Dicle University Medical Faculty Hospital. Out of an initial 1,800 records identified between January 2024 and October 2025, a final cohort of 128 patients met the inclusion criteria for the analysis of inflammatory disease activity, sleep quality, and health-related quality of life.

Data for the RA cohort were obtained via a retrospective review of electronic medical records from the Rheumatology Department of Dicle University Hospital. Between January 2024 and October 2025, 1,800 patients were initially screened. After excluding those under 18 years of age, with incomplete records, or who did not meet the diagnostic and exclusion criteria, a total of 128 RA patients were included in the final analysis (Figure 1). The number of healthy controls (*n* = 115) was slightly lower than that of RA patients due to the strict inclusion and exclusion criteria applied to ensure comparability with the RA cohort. Controls with any history of autoimmune, inflammatory, or chronic systemic diseases, recent infections, or use of sleep-affecting medications were excluded. Recruitment was also limited to individuals who completed all required assessments. This careful selection was prioritized over matching the numbers exactly, to reduce potential confounding and maintain the validity of group comparisons.

The study protocol was approved by the Dicle University Institutional Ethics Committee (Approval No: 2025/400), and all procedures adhered to the principles of the Declaration of Helsinki. Due to the retrospective and anonymized nature of the dataset, the requirement for informed consent was waived by the ethics committee.

### Patient and public involvement

Patients and the public were not involved in the design, conduct, reporting, or dissemination plans of this research.

### Clinical and anthropometric data acquisition

Demographic information, including age, sex, marital status, education level, and smoking status, was systematically extracted from electronic medical records. Anthropometric measurements—height, weight, and waist and hip circumferences—were obtained by trained clinical staff following standardized protocols. Body weight was measured to the nearest 0.1 kg and height to the nearest 0.5 cm, with participants wearing light clothing and no shoes. Waist circumference was measured at the midpoint between the lower rib margin and the iliac crest, and hip circumference at the widest point over the buttocks, in accordance with World Health Organization (WHO) guidelines. These measurements were used to calculate validated indices of body composition and metabolic risk, including body mass index (BMI; weight in kilograms divided by the square of height in meters, kg/m^2^), waist-to-hip ratio (WHR; waist circumference divided by hip circumference), and waist-to-height ratio (WHtR; waist circumference divided by height), with WHtR serving as a marker of central adiposity and cardiometabolic risk. All assessments were conducted during the same clinical visit to ensure data consistency and reproducibility.

### Laboratory assessments

A comprehensive panel of laboratory parameters was extracted from institutional medical records, corresponding to the same clinical visit as the anthropometric and survey assessments. Hematological parameters—including white blood cell count (WBC), hemoglobin (Hgb), absolute lymphocyte count (LYM), and platelet count (PLT)—were measured using an automated hematology analyzer. Systemic inflammation was assessed via ESR and CRP levels. Serum 25-hydroxyvitamin D (Vitamin D status was categorized as deficiency <20 ng/mL, insufficiency 20–30 ng/mL, and sufficiency >30 ng/mL), ferritin, and Vitamin B12 levels were determined using standard electrochemiluminescence immunoassay (ECLIA) protocols (18).

To evaluate the interplay between systemic inflammation and metabolic dysregulation, several validated composite indices were calculated. These included the NLR, PLR, TyG index {calculated as ln [fasting triglycerides (mg/dL) × fasting glucose (mg/dL)/2], a surrogate marker of insulin resistance, CMI; [triglycerides/HDL-C] × waist-to-height ratio, integrating lipid profile and central adiposity}, plasma atherogenic index [PAI; log₁₀ (triglycerides/HDL-C)], and monocyte-to-HDL ratio (MHR; monocyte count divided by HDL-cholesterol). The selection of these markers was based on their established relevance to systemic inflammation, metabolic stress, and potential impact on sleep quality and HRQoL in RA, complementing conventional inflammatory markers (ESR, CRP). All laboratory analyses were performed by the institutional central laboratory following standardized internal and external quality control procedures.

### Clinical assessments and patient-reported outcomes

RA disease activity was assessed using the Disease Activity Score in 28 joints (DAS28-ESR), a validated composite index that integrates tender joint count (TJC), swollen joint count (SJC), the patient’s global health assessment (visual analog scale, 0–100 mm), and ESR (19). DAS28 scores were classified as remission (<2.6), low (2.6–3.2), moderate (3.2–5.1), and high (>5.1) disease activity.

Subjective sleep quality was evaluated using validated Turkish version of the PSQI, which assesses sleep disturbances over the preceding month across seven components: subjective sleep quality, sleep latency, sleep duration, habitual sleep efficiency, sleep disturbances, use of sleeping medication, and daytime dysfunction. Total PSQI scores range from 0 to 21, with higher scores indicating poorer sleep quality; a global score >5 is typically used to distinguish poor sleepers from good sleepers (20, 21).

HRQoL was measured using validated Turkish version of the SF-36 questionnaire, which evaluates eight domains: Physical Functioning, Role Physical, Bodily Pain, General Health, Vitality, Social Functioning, Role Emotional, and Mental Health. Scores for each domain range from 0 to 100, with higher scores reflecting better health status and quality of life (22, 23). All patient-reported outcome assessments were conducted by the same clinical team to ensure consistency and minimize inter-rater variability.

### Statistical analysis

All statistical analyses were performed using IBM SPSS Statistics version 27.0 (IBM Corp., Armonk, NY, USA). A *post hoc* power analysis indicated that the final sample size provided >80% power at *α* = 0.05 to detect medium effect sizes (f^2^ = 0.15) for the primary regression models, ensuring adequate statistical robustness. Continuous variables are presented as means ± standard deviations (SDs) and were compared using independent samples t-tests for normally distributed data, while non-normally distributed variables were analyzed using the Mann–Whitney U test after confirming normality with the Kolmogorov–Smirnov test. Categorical variables are expressed as frequencies (percentages) and compared using Chi-square tests. Spearman’s rank correlation coefficients (*ρ*) were calculated to examine associations between clinical, inflammatory (ESR, CRP), and metabolic (TyG, CMI) parameters and outcomes (PSQI total and component scores, SF-36). Variables demonstrating significant univariate correlations were subsequently entered into stepwise multiple linear regression models to identify independent predictors of sleep quality, with multicollinearity assessed using the variance inflation factor (VIF), where values <5 were considered acceptable. Regression models were adjusted for potential confounders including age, sex, disease duration, use of biologic therapy, glucocorticoid therapy, and psychiatric comorbidities.

To test the hypothesis that sleep quality (PSQI) mediates the relationship between disease activity (DAS28-ESR) and HRQoL (SF-36), mediation analysis was conducted using the PROCESS macro for SPSS (Model 4, developed by Andrew F. Hayes) (24). The indirect effect was estimated using a percentile bootstrapping procedure with 5,000 resamples, and a 95% confidence interval (CI) that did not include zero was considered indicative of a statistically significant mediation effect. Separate exploratory analyses were also performed for individual PSQI component scores to evaluate their specific contribution to the mediation pathway. Across all analyses, two-tailed *p* < 0.05 were considered statistically significant.

**Figure 2:**
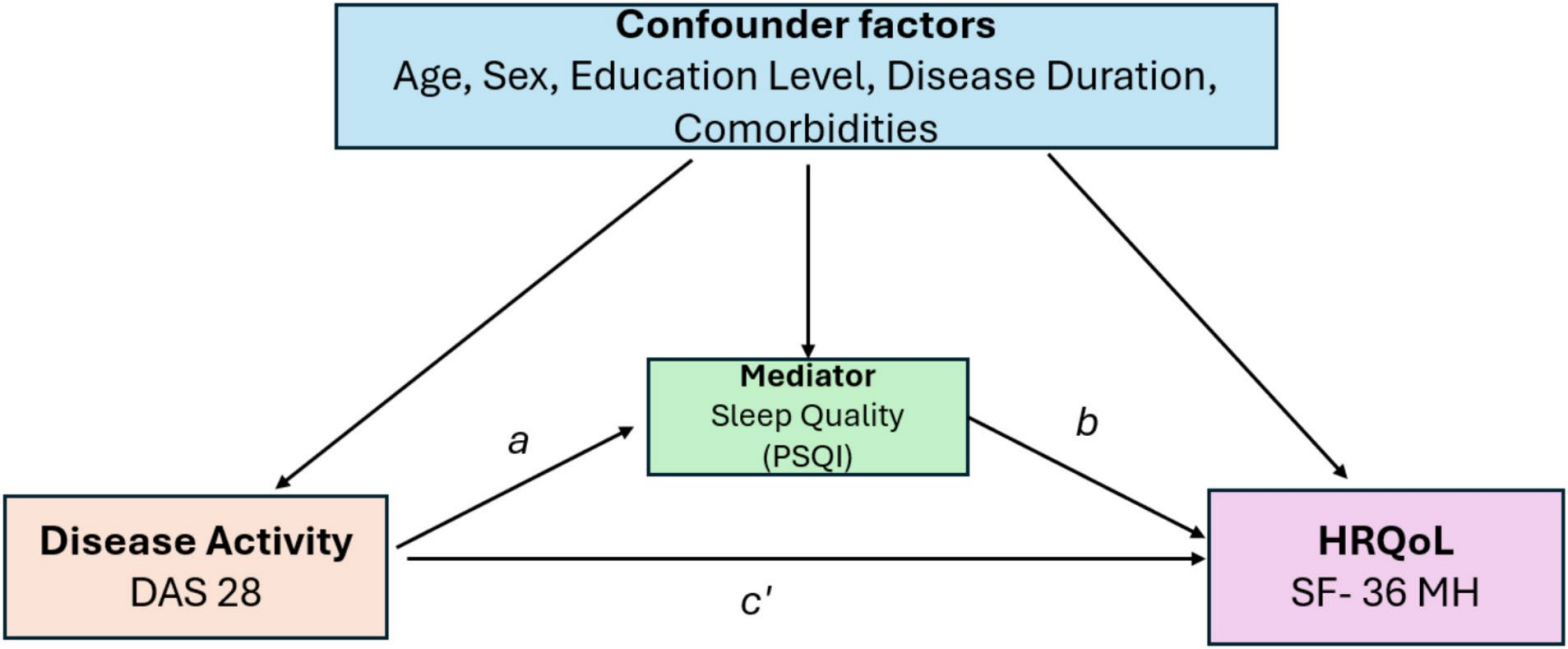
Directed acyclic graph illustrating the mediation model in which sleep quality (PSQI) mediates the association between disease activity (DAS28) and mental health (SF-36 mental health domain). The total effect of DAS28 on mental health was statistically significant (*c* = −5.82, *p* < 0.001). The indirect effect of DAS28 on mental health through sleep quality was also statistically significant [indirect effect = −1.42, BootSE = 0.45, 95% CI (−2.38, −0.62)]. After the inclusion of sleep quality as a mediator, the direct effect of DAS28 on mental health remained statistically significant but was attenuated *c* prime (*c′*), indicating partial mediation. Age, sex, education level, disease duration, and comorbidities were included as covariates in the model (paths not shown for simplicity). DAS28, Disease Activity Score in 28 joints; PSQI, Pittsburgh Sleep Quality Index; SF-36 MH, Short Form-36 Mental Health.

To formally test whether sleep quality mediates the relationship between disease activity and health-related quality of life, a mediation model was specified (Figure 2). Disease activity (DAS28-ESR) was defined as the independent variable, sleep quality (PSQI total score) as the mediator, and mental health (SF-36 MH) as the outcome.

## Results

The study cohort consisted of 128 patients with rheumatoid arthritis (RA) and 115 age- and sex-matched healthy controls. The groups were well-balanced in terms of age (RA: 51.10 ± 13.23 years; Controls: 50.73 ± 13.17 years; *p* = 0.827), sex distribution (74.2% female in RA vs. 73.9% in controls; *p* = 0.763), and body mass index (BMI) (RA: 27.35 ± 5.66 kg/m^2^; Controls: 26.94 ± 4.43 kg/m^2^; *p* = 0.794). Significant differences emerged in socioeconomic and lifestyle factors, with a markedly higher proportion of university graduates observed among controls compared to RA patients (51.3% vs. 9.4%; *p* < 0.001), while current smoking was more prevalent in the control group (63.5% vs. 29.9%; *p* < 0.001). Marital status did not differ significantly between groups (*p* = 0.099). To mitigate potential confounding effects, education level and smoking status were adjusted for in subsequent multivariable analyses.

RA patients exhibited significantly elevated markers of systemic inflammation compared with healthy controls. Hematological analyses revealed higher white blood cell (WBC) counts (8.42 × 10^3^/μL vs. 6.57 × 10^3^/μL; *p* < 0.001), platelet counts (299.34 × 10^3^/μL vs. 224.73 × 10^3^/μL; *p* < 0.001), erythrocyte sedimentation rate (ESR; 27.23 ± 16.11 vs. 7.59 ± 3.51 mm/h; *p* < 0.001), and ferritin levels (234.92 vs. 81.57 ng/mL; *p* < 0.001) in the RA group. In contrast, hemoglobin levels were lower in RA patients (12.87 vs. 13.56 g/dL; *p* = 0.001), consistent with anemia of chronic disease.

Markers of systemic inflammation and immune dysregulation, including the neutrophil-to-lymphocyte ratio (NLR: 2.50 vs. 1.95) and platelet-to-lymphocyte ratio (PLR: 144.21 vs. 106.80), were also significantly elevated in RA patients (*p* < 0.001 for both). Furthermore, the TyG index, a surrogate marker of insulin resistance, was higher in the RA group (8.75 vs. 8.42; *p* < 0.001).

Anthropometric assessments indicated greater visceral adiposity in RA patients, reflected by increased waist circumference (92.39 vs. 84.43 cm; *p* < 0.001) and waist-to-hip ratio (0.57 vs. 0.52; *p* < 0.001). Critically, serum Vitamin D levels were significantly lower in RA patients (18.98 vs. 31.36 ng/mL; *p* < 0.001). No significant differences were observed for Vitamin B₁₂, monocyte-to-HDL ratio (MHR), plasma atherogenic index (PAI), or cardiometabolic index (CMI) (*p* > 0.05).

Patient-reported outcomes demonstrated a substantial burden of sleep disturbance and impaired quality of life in the RA cohort. RA patients exhibited significantly poorer sleep quality compared with healthy controls, as reflected by higher global PSQI scores (8.03 ± 3.97 vs. 4.14 ± 3.09; *p* < 0.001). Regarding health-related quality of life, RA patients showed significant impairment across multiple SF-36 domains. The most pronounced differences were observed in the Mental Health (35.94 ± 17.96 vs. 68.12 ± 17.12; *p* < 0.001) and General Health (42.50 ± 20.26 vs. 64.80 ± 14.52; *p* < 0.001) domains. Additionally, scores were significantly lower in the Role Physical (*p* = 0.014) and Role Emotional (*p* < 0.001) subscales, indicating the impact of RA on both physical and psychological functioning. In contrast, no significant differences were detected for Physical Functioning, Bodily Pain, Vitality, or Social Functioning (all *p* > 0.05), suggesting that, in this cohort, the perceived burden of RA was predominantly centered on mental health and role-related limitations.

**Figure 3:**
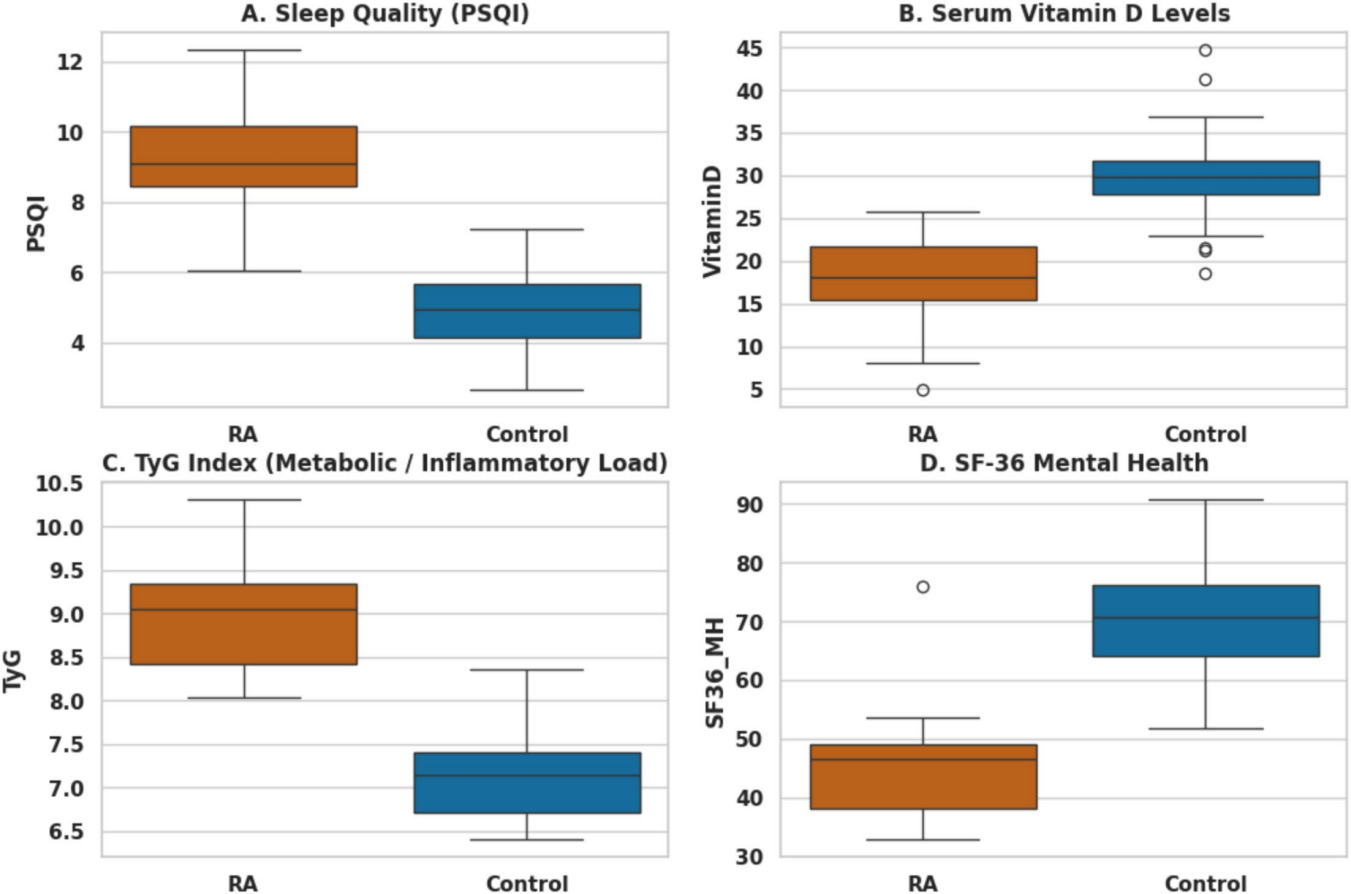
Multidimensional comparison of patient-reported outcomes and bio-metabolic profiles between rheumatoid arthritis (RA) patients and healthy controls. Panels illustrate significant group differences in: **(A)** subjective sleep quality (PSQI), where higher scores indicate greater impairment; **(B)** serum vitamin D levels, highlighting the prevalent deficiency in RA; **(C)** metabolic burden assessed by the Triglyceride–Glucose (TyG) index; and **(D)** mental health-related quality of life (SF-36 MH). Box plots represent the median and interquartile range (IQR), with whiskers extending to 1.5 × IQR. Significant differences between RA patients and controls were observed across all panels (*p* < 0.001). Abbreviations: PSQI, Pittsburgh Sleep Quality Index; TyG, Triglyceride–Glucose Index; SF-36 MH, Short Form-36 Mental Health.

Table 1 presents the baseline demographic, clinical, and key biochemical characteristics of the study groups. RA patients exhibited significantly poorer sleep quality, lower serum vitamin D levels, and a higher metabolic burden compared with healthy controls (Figures 3A–C). These bio-metabolic alterations were accompanied by a marked reduction in mental health-related quality of life (Figure 3D), with all differences reaching statistical significance (*p* < 0.001).

**Table 1:** Baseline demographic, clinical, and biochemical characteristics of the study groups.

Parameter	RA group (*n* = 128)	Control group (*n* = 115)	*p*-value
Demographic characteristics			
Age (years)	51.10 ± 13.23	50.73 ± 13.17	0.827
BMI (kg/m^2^)	27.35 ± 5.66	26.94 ± 4.43	0.794
University education, n (%)	12 (9.4)	59 (51.3)	<0.001
Current smoker, n (%)	38 (29.9)	73 (63.5)	<0.001
Patient-reported outcomes (PROs)			
PSQI (sleep quality)	8.03 ± 3.97	4.14 ± 3.09	<0.001
SF-36 mental health	35.94 ± 17.96	68.12 ± 17.12	<0.001
SF-36 general health	42.50 ± 20.26	64.80 ± 14.52	<0.001
Inflammatory and metabolic markers			
ESR (mm/h)	27.23 ± 16.11	7.59 ± 3.51	<0.001
CRP (mg/L)	14.98 ± 16.19	7.80 ± 5.09	<0.001
NLR	3.24 ± 2.04	1.95 ± 0.52	<0.001
TyG index	8.75 ± 0.66	8.42 ± 0.41	<0.001
Vitamin D (ng/mL)	17.75 ± 9.82	31.36 ± 6.70	<0.001

Data are presented as mean ± standard deviation or number (percentage).

Continuous variables were compared using Student’s *t*-test or Mann–Whitney U test according to data distribution, while categorical variables were analyzed using the χ^2^ test. BMI, body mass index; PSQI, Pittsburgh Sleep Quality Index; SF-36, Short Form-36 Health Survey; ESR, erythrocyte sedimentation rate; CRP, C-reactive protein; NLR, neutrophil-to-lymphocyte ratio; TyG, triglyceride–glucose index; RA, rheumatoid arthritis.

Spearman’s rank correlation analyses revealed several significant associations between clinical, metabolic, and patient-reported outcomes (Table 2). Disease activity, as measured by DAS28, was strongly positively correlated with poorer sleep quality (PSQI: *ρ* = 0.360, *p* < 0.001) and inversely associated with all SF-36 domains, particularly General Health (ρ = −0.432) and Mental Health (*ρ* = −0.319; both *p* < 0.001), highlighting the central role of systemic disease activity in driving both psychological and physical impairment.

**Table 2:** Spearman correlation analysis between disease activity (DAS28) and clinical, inflammatory, metabolic, and patient-reported outcome variables.

Category	Variable	Spearman’s *ρ*	*p*-value
Clinical	DAS28	0.360	<0.001
Inflammatory	ESR	0.258	0.003
	CRP	0.195	0.030
Metabolic	Vitamin D	−0.213	0.012
	TyG index	0.189	0.046
	Cardiometabolic index	0.199	0.026
Patient-reported outcomes	SF-36 mental health	−0.342	<0.001
	SF-36 general health	−0.325	<0.001

Spearman’s rank correlation coefficient (ρ) was used to assess associations between DAS28 and continuous variables. DAS28, Disease Activity Score in 28 joints; ESR, erythrocyte sedimentation rate; CRP, C-reactive protein; SF-36, Short Form-36 Health Survey; TyG, triglyceride–glucose index.

Exploratory analyses of individual PSQI components were conducted to further characterize which dimensions of sleep were most affected by disease activity. The strongest associations were observed for sleep latency, sleep disturbances, and daytime dysfunction, suggesting that these aspects contribute most substantially to overall sleep impairment in RA patients (Supplementary Table S1). Specifically, sleep latency (*ρ* = 0.34, *p* < 0.001), sleep disturbances (ρ = 0.31, *p* < 0.001), and daytime dysfunction (*ρ* = 0.36, *p* < 0.001) were positively correlated with DAS28, whereas other components, such as sleep duration, habitual sleep efficiency, subjective sleep quality, and use of sleeping medication, demonstrated weaker or non-significant correlations.

Key inflammatory markers, including ESR (*ρ* = 0.258, *p* = 0.003) and CRP (ρ = 0.195, *p* = 0.030), were significantly associated with higher PSQI scores, indicating that systemic inflammation contributes to sleep disturbance. Metabolic indices also demonstrated notable correlations with sleep quality, with waist circumference (ρ = 0.212, *p* = 0.016), TyG index (ρ = 0.189, *p* = 0.046), and Cardiometabolic Index (ρ = 0.199, *p* = 0.026) all positively correlated with PSQI scores.

Serum Vitamin D levels were inversely associated with both DAS28 (ρ = −0.400, *p* < 0.001) and PSQI scores (ρ = −0.213, *p* = 0.012), suggesting a dual modulatory role in RA by influencing both the inflammatory cascade and sleep architecture.

To identify independent determinants of subjective sleep quality in RA patients, a multivariable linear regression model was constructed (Table 3). The overall model was highly significant (*F* = 28.42, *p* < 0.001) and explained 53% of the variance in PSQI scores (*R*^2^ = 0.53; adjusted *R*^2^ = 0.51). Disease activity, measured by DAS28, emerged as the strongest independent predictor of impaired sleep quality (*β* = 0.534, *p* < 0.001). In addition, systemic inflammatory markers—ESR (*β* = 0.273, *p* < 0.001) and CRP (*β* = 0.212, *p* < 0.001)—remained significant predictors after adjusting for metabolic factors. Serum Vitamin D was identified as a protective factor, with higher levels independently associated with better sleep quality (*β* = −0.173, *p* = 0.002), suggesting that Vitamin D deficiency contributes directly to sleep disturbance irrespective of inflammatory burden.

**Table 3:** Multivariable linear regression analysis identifying independent predictors of sleep quality (PSQI) in RA (*N* = 128).

Predictor	Standardized β	Unstandardized B (SE)	*p*-value	VIF
Disease activity (DAS28)	0.534	1.65 (0.18)	<0.001	1.38
ESR (mm/h)	0.273	0.07 (0.01)	<0.001	1.26
CRP (mg/L)	0.212	0.05 (0.01)	<0.001	1.22
Vitamin D (ng/mL)	−0.173	−0.07 (0.02)	0.002	1.22
NLR	−0.072	−0.14 (0.11)	0.191	1.23
TyG index	0.051	0.31 (0.59)	0.604	3.90
Cardiometabolic index (CMI)	0.109	<0.001 (0.00)	0.275	3.99

Model Statistics: *F* = 28.42, *p* < 0.001, *R*^2^ = 0.53, Adjusted *R*^2^ = 0.51, B = unstandardized coefficient; β = standardized coefficient; SE, standard error; VIF, variance inflation factor; DAS28, Disease Activity Score in 28 joints; ESR, erythrocyte sedimentation rate; CRP, C-reactive protein; NLR, neutrophil-to-lymphocyte ratio; TyG, triglyceride-glucose index; WHtR, waist-to-height ratio; CMI, cardiometabolic index.

Other variables that showed significant univariate correlations, including NLR, TyG index, Cardiometabolic Index, and WHtR, did not retain significance in the multivariable model (all *p* > 0.05), indicating that metabolic dysfunction may influence sleep indirectly or via systemic inflammation and disease activity. Multicollinearity was not a concern, with all variance inflation factor (VIF) values below 5 (range: 1.180–3.993).

The mediation framework conceptualized in Figure 2 supports sleep quality as a functional bridge linking inflammatory disease activity to mental health impairment. To further explore the pathways linking disease activity to patient well-being, mediation analyses were conducted to assess whether sleep quality (PSQI) mediates the relationship between DAS28 and the physical and mental health domains of HRQoL (SF-36). The total effect of DAS28 on both Mental Health (*c* = −5.82, *p* < 0.001) and General Health (*c* = −4.15, *p* < 0.001) was significant. After including sleep quality as a mediator, the direct effects of DAS28 on these outcomes remained statistically significant but were attenuated, indicating that sleep quality explains a substantial portion of the relationship.

Critically, the indirect effects through PSQI were significant for both domains: Mental Health [Indirect Effect = −1.42, BootSE = 0.45, 95% CI (−2.38, −0.62)] and General Health (Indirect Effect = −0.98, BootSE = 0.32, 95% CI [−1.75, −0.45]). As the confidence intervals did not include zero, sleep quality was confirmed as a significant partial mediator. These findings suggest that disrupted sleep transmits a meaningful portion of the adverse impact of RA disease activity on both physical and psychological aspects of quality of life, underscoring sleep as a potential therapeutic target to improve patient-centered outcomes. The coefficients and standard errors for the mediation pathways are detailed in Table 4.

**Table 4:** Mediation analysis of sleep quality (PSQI) as a mediator between disease activity (DAS28) and HRQoL domains (SF-36).

HRQoL domain	Effect type	Coefficient/effect (*β*)	SE (boot)	95% CI (LL, UL)	*p*-value
Mental health	Total effect (c)	−5.82	0.58	[−6.96, −4.68]	<0.001
	Direct effect (c’)	−4.40	0.62	[−5.62, −3.18]	<0.001
	Indirect effect (a × b)	−1.42	0.45	[−2.38, −0.62]	–
General health	Total Effect (c)	−4.15	0.42	[−4.98, −3.32]	<0.001
	Direct Effect (c’)	−3.17	0.48	[−4.12, −2.22]	<0.001
	Indirect Effect (a × b)	−0.98	0.32	[−1.75, −0.45]	–

SE (Boot), Bootstrapped Standard Error; CI, Confidence Interval; LL, Lower Limit; UL, Upper Limit. Indirect effects were estimated using 5,000 bootstrap resamples. A 95% CI that does not cross zero indicates a statistically significant mediation effect.

The mediation analysis revealed that sleep quality (PSQI) significantly mediated the relationship between disease activity (DAS28) and HRQoL. Specifically, PSQI accounted for 24.4% of the total effect of DAS28 on Mental Health [Indirect Effect = −1.42, 95% CI (−2.38, −0.62)] and 23.6% of the effect on General Health [Indirect Effect = −0.98, 95% CI (−1.75, −0.45)]. Since the 95% confidence intervals did not include zero, the mediating role of sleep quality was statistically significant. To further quantify the impact of sleep quality, the proportion of the effect mediated by PSQI was calculated and is presented in Table 5.

**Table 5:** Mediation analysis of sleep quality (PSQI) in the association between disease activity (DAS28) and health-related quality of life (SF-36).

SF-36 domain	Total effect (c) coefficient (SE)	Direct effect (c′) coefficient (SE)	Indirect effect (ab), effect (95% CI)	Mediation ratio (%)	*p*-value
Mental health	−5.82 (0.58)	−4.40 (0.62)	−1.42 (−2.38 to −0.62)	24.4	<0.001
General health	−4.15 (0.42)	−3.17 (0.48)	−0.98 (−1.75 to −0.45)	23.6	<0.001

Independent Variable (X): Disease Activity Score-28 (DAS28) Mediator (M): Sleep Quality assessed by the Pittsburgh Sleep Quality Index (PSQI) Dependent Variables (Y): SF-36 Mental Health and General Health domains. Total Effect (c) represents the overall association between DAS28 and HRQoL domains. Direct Effect (c′) represents the association after adjusting for sleep quality. Indirect Effect (ab) reflects the proportion of the association mediated through sleep quality. Mediation Ratio was calculated as (Indirect Effect/Total Effect) × 100. Indirect effects and 95% confidence intervals (CI) were estimated using 5,000 bootstrap resamples. Statistical significance of mediation was inferred when the 95% CI did not include zero.

Sensitivity analyses confirmed the robustness of the mediating role of sleep quality across sex and key metabolic and socioeconomic covariates. Furthermore, interaction analyses demonstrated that poor sleep quality significantly amplified the adverse effect of disease activity on mental health, indicating a synergistic rather than merely additive relationship (Table 6).

**Table 6:** Sensitivity and interaction analyses examining the robustness of the mediating role of sleep quality (PSQI) in the association between disease activity (DAS28) and mental health (SF-36).

Model component	Original estimate	Sensitivity A	Sensitivity B	Sensitivity C
Total effect (DAS28 → mental health)	−5.82	−5.70	−5.85	−5.80
Direct effect (adjusted for PSQI)	−4.40	−4.35	−4.42	−4.38
Interaction model				
DAS28	−4.20	−4.15	−4.22	−4.18
PSQI	−1.15	−1.10	−1.16	−1.12
DAS28 × PSQI	1.35*	1.40*	1.30*	1.32*

Sensitivity A: Analysis restricted to female participants (*n* = 95), Sensitivity B: Model additionally adjusted for smoking status, Sensitivity C: Model additionally adjusted for education level, Values are presented as unstandardized regression coefficients, *p < 0.05 for interaction terms. A significant DAS28 × PSQI interaction indicates that poorer sleep quality amplifies the negative impact of disease activity on mental health outcomes.

## Discussion

The present study provides a comprehensive and integrative evaluation of the complex interplay between systemic inflammation, metabolic dysregulation, sleep quality, and HRQoL in patients with RA. While impaired sleep quality and reduced quality of life are well-recognized features of RA, the mechanistic pathways through which disease activity translates into patient-perceived morbidity have remained incompletely understood (5, 25). The principal and most novel contribution of this work lies in the formal quantification of sleep quality as a mediating mechanism, demonstrating that approximately one-quarter of the detrimental effect of disease activity on HRQoL is transmitted through sleep disruption. These findings reposition sleep not as a secondary symptom but as a functional biological and clinical bridge linking inflammatory disease activity to impaired physical and mental well-being.

A central finding of our study is the robust and consistent association between heightened systemic inflammation and impaired sleep quality in RA. Both classical inflammatory markers—ESR and CRP—and composite hematological indices reflecting immune imbalance, including the NLR and PLR, were significantly correlated with poorer sleep quality as measured by PSQI. Notably, NLR and PLR demonstrated an approximately 1.6-fold elevation in RA patients compared with healthy controls, highlighting the magnitude of chronic immune activation.

These findings suggest that sleep disturbance in RA is not merely a secondary consequence of mechanical joint pain or nocturnal discomfort, but rather an intrinsic manifestation of systemic immune-inflammatory dysregulation. This observation aligns with previous studies reporting a high prevalence of insomnia and non-restorative sleep in RA and its association with disease burden and functional impairment (8, 26, 27). Our study further extends the literature by emphasizing that composite inflammatory indices, which capture the balance between innate and adaptive immunity, may offer a more stable and clinically informative reflection of the inflammatory milieu driving sleep disruption than single-point acute-phase reactants alone.

From a mechanistic perspective, the intimate crosstalk between the immune system and sleep regulation provides a plausible biological substrate for our observations. Pro-inflammatory cytokines—particularly tumor necrosis factor-*α* (TNF-α), interleukin-6 (IL-6), and interleukin-1β—are markedly elevated in RA and play a dual role in immune activation and sleep modulation (28, 29). These cytokines can cross the blood–brain barrier or signal through afferent vagal pathways to influence hypothalamic and brainstem sleep centers, resulting in reduced slow-wave sleep, increased nocturnal arousals, and fragmented sleep architecture (30).

Experimental and clinical studies further demonstrate that sleep disturbance itself amplifies inflammatory signaling, establishing a bidirectional and self-perpetuating cycle between inflammation and sleep fragmentation (31–33). In our cohort, inflammatory markers remained significantly associated with impaired sleep quality even after adjustment for overall disease activity, suggesting that residual systemic inflammation independently disrupts sleep regulation.

Beyond inflammation, metabolic stress emerged as an important contributor to sleep impairment in our RA cohort. Measures of visceral adiposity and insulin resistance—including WHtR, TyG index, and the CMI—were positively correlated with PSQI scores (34–36). These associations suggest a synergistic interaction in which metabolic dysfunction potentiates the deleterious effects of systemic inflammation on sleep (37).

Adipose tissue is now recognized as an active endocrine organ, secreting a range of pro-inflammatory adipokines (e.g., leptin, resistin) and cytokines, including IL-6 and TNF-*α*, which can further amplify systemic immune activation (38). In parallel, insulin resistance has been linked to disturbances in circadian rhythm regulation and dysregulation of neurohormonal systems, particularly melatonin and cortisol, both critical for sleep initiation and maintenance (39).

Importantly, our multivariable regression analyses clarified that metabolic indices did not retain independent predictive value for sleep quality once disease activity and inflammatory markers were accounted for. This suggests that metabolic dysfunction may act as an amplifier of inflammation-driven sleep disruption rather than as a primary driver, reinforcing a multidimensional model in which systemic inflammation remains central.

A particularly compelling aspect of our findings is the identification of serum Vitamin D status as an independent predictor of sleep quality in RA. RA patients exhibited markedly lower Vitamin D levels than healthy controls, and Vitamin D was inversely correlated with both disease activity and PSQI scores. This association persisted after adjustment for inflammatory and metabolic factors, highlighting a direct, independent role of Vitamin D in sleep regulation.

Vitamin D functions as a negative regulator of the inflammatory cascade, suppressing pro-inflammatory cytokines such as TNF-*α*, IL-6, and IL-1β, and also influences neuro-hormonal pathways relevant to sleep (40, 41). It participates in the conversion of tryptophan to serotonin, the precursor of melatonin, which governs circadian rhythm and sleep maintenance (42). Vitamin D receptors are expressed in key sleep–wake regulatory regions of the brain, providing a plausible neurobiological substrate for its effect on sleep architecture (40).

The most distinctive and novel contribution of this study lies in the formal demonstration that sleep quality partially mediates the relationship between RA disease activity and HRQoL. Sleep quality accounted for 24.4% of the total effect of DAS28 on Mental Health and 23.6% on General Health, with robust 95% confidence intervals that did not cross zero, confirming statistical significance. This quantification moves beyond descriptive associations, positioning sleep as a mechanistic intermediate translating inflammatory burden into psychological and physical impairment (43).

From a clinical perspective, these findings carry important implications for RA management (44). Current T2T strategies prioritize inflammatory remission and joint counts, often achieving improvements in objective disease measures. However, our data indicate that nearly one-quarter of patient-perceived morbidity may remain unresolved due to persistent sleep disruption (45). Incorporating systematic sleep assessment and management strategies—such as sleep hygiene education, CBT-I, Vitamin D optimization, and targeted pain control—into existing T2T frameworks may help address this residual burden (46, 47).

Our analysis also accounted for potential confounders. Despite higher smoking rates and lower educational attainment in the control group, RA patients displayed markedly poorer sleep quality and higher inflammatory burden, reinforcing the dominant role of RA-specific inflammatory pathways over lifestyle factors in driving sleep disturbance. Sensitivity analyses confirmed that the mediating role of sleep remained robust across sex, smoking status, and education level.

Strengths of the study include its integrative design, simultaneous assessment of inflammatory, metabolic, endocrine, and patient-reported domains, formal mediation analysis with bootstrap resampling, inclusion of a well-matched control group, and use of globally validated instruments.

Several limitations warrant consideration. The cross-sectional design precludes definitive causal inference, and the bidirectional nature of the inflammation–sleep axis suggests that longitudinal studies are needed. Sleep quality was assessed via PSQI, a subjective measure; no polysomnography or actigraphy was performed. The single-center design may limit generalizability, and unmeasured confounders—such as specific medications, psychological comorbidities, or physical activity—should be addressed in future research.

Future studies should adopt longitudinal and interventional designs to determine whether improving sleep quality can actively attenuate disease activity and accelerate HRQoL recovery. Objective sleep assessments and exploration of molecular pathways—including adipokines, circadian rhythm genes (e.g., CLOCK, PER2), and neuroimmune signaling—may identify novel therapeutic targets at the intersection of inflammation, metabolism, and sleep.

## Conclusion

In summary, this study provides robust evidence that systemic inflammation, metabolic dysregulation—characterized by insulin resistance and visceral adiposity—and Vitamin D deficiency collectively contribute to significant sleep impairment and reduced quality of life in patients with Rheumatoid Arthritis. Our findings identify disease activity (DAS28) and systemic inflammatory markers as the principal independent predictors of sleep disruption, with Vitamin D status exerting a distinct, protective effect. Importantly, mediation analysis demonstrates that sleep quality functions as a critical intermediary, partially transmitting the detrimental impact of joint inflammation on both mental and general health. These results underscore the presence of a complex, integrated pathophysiological network in RA that extends beyond localized joint involvement. Taken together, our findings support a holistic, patient-centered management approach that may benefit from not only inflammatory control but also attention to metabolic health, Vitamin D optimization, and sleep quality, with the potential to improve the physical and psychological well-being of individuals living with RA.

## Data Availability

The data analyzed in this study is subject to the following licenses/restrictions: The dataset generated and analyzed during the current study is not publicly available due to ethical and privacy considerations, as it contains sensitive clinical and personal health information that could potentially identify individual participants. Access to anonymized data may be granted upon reasonable request to the corresponding author, subject to approval by the institutional ethics committee and in accordance with applicable data protection regulations. Requests to access these datasets should be directed to Zeynel Abidin Akar, zeynelabidin_akar@yahoo.com.
