# Supplementary material for: The mediating role of sleep quality in the association between inflammatory disease activity and health-related quality of life in rheumatoid arthritis
Source: Front Med (Lausanne). 2026 Mar 16;13:1797652. doi: 10.3389/fmed.2026.1797652 (PMC13033770; doi:10.3389/fmed.2026.1797652)
Supplement: Supplementary file 1 [file Table_1.docx]

**Supplementary Table S1:** Exploratory correlations between PSQI component scores and DAS28 in RA patients

| **PSQI Component** | **Spearman ρ** | **p-value** |
| --- | --- | --- |
| Subjective sleep quality | 0.21 | 0.021 |
| Sleep latency | 0.34 | <0.001 |
| Sleep duration | 0.12 | 0.145 |
| Habitual sleep efficiency | 0.18 | 0.049 |
| Sleep disturbances | 0.31 | <0.001 |
| Use of sleeping medication | 0.15 | 0.080 |
| Daytime dysfunction | 0.36 | <0.001 |

Correlations were assessed using Spearman’s rank correlation coefficient (ρ). Significant correlations (p < 0.05) are highlighted. The strongest associations were observed for sleep latency, sleep disturbances, and daytime dysfunction, indicating these domains of PSQI are most closely linked to disease activity (DAS28).
